# Colloid Tumor of the Broad Ligament, Etc.

**Published:** 1870-06

**Authors:** John E. Owens

**Affiliations:** Surgeon to St. Luke’s Hospital; No. 112 Randolph Street


A MONTHLY RECORD OF

*Medicine, Surgery, and the Collateral Sciences.*

EDITED BY J. ADAMS ALLEN, M.D., LL.D.; AND WALTER HAY, M.D.

VOL. XXVII.—JUNE, 1870.—No. 6.

### Original Communications.

ARTICLE I.—*Colloid Tumor of the Broad Ligament; Two Uterine Tumors; Persistent Ascites; Gastrotomy, Recovery; Subsequent Pregnancy, and Parturition of Still-Born Child.*

(Under the care of JNO. E. OWENS, M. D., Surgeon to St. Luke's Hospital.)

Mrs. F., æt. 25, well-nourished, florid complexion, came under my care, August 26, 1867, eight months previous to which she was married. She had never been pregnant. Menstruation, which commenced when she was 17 or 18 years of age, continued normally for two years, since which time it has been irregular, very scanty, and, during the last year, very painful. Her last menstruation occurred about six weeks ago, there being but very little blood. Urination has not been normal since last December—sometimes passing only a half-teacupful, and at others nearly a quart. In the spring of 1866, she discovered a tumor in the left iliac region, the size of a small orange. When first seen, she presented the appearance of a woman advanced to the 8th or 9th month of pregnancy. It was on account of this tumor and of the distended abdomen, which interfered with respiration, digestion and locomotion, that she sought advice. She was the more solicitous for advice, because a medical gentleman had diagnosed preg nancy, and the ordinary limit of a physiological tumor had passed. Suffice it to say, that upon tapping the abdomen, a rather heavy tumor principally to the left of the median line, and reaching as far as the umbilicus, was discovered. At a subsequent tapping, a small tumor was made out, growing, it was supposed, from the uterus. There was quite prominent a fluid umbilical hernia, whose walls, later in the history of the case, were not much thicker than parchment. Profs. Byford and Powell were good enough to see this case with me. In the first months of the tappings, an operation seemed to be contraindicated, because of the following reasons:—The mobility of the tumor was not then distinctly made out,—an operation was not urgent,—therapeutic means of disposing of the fluid had not been tried. The contraindications, however, by degrees, subsided, viz:—The tumor was found to be quite movable, operative interference became urgent,—therapeutic means failed. Diuretics effected no increase in the quantity of urine. Elaterium produced copious watery discharges, but the slight reduction in the size of the abdomen was far out of proportion to the distress and debility occasioned by the action of the medicine. The patient was reluctant to submit to its further use, but strenuously insisted upon an operation. That the case was an urgent one, may be gleaned, in part, from the following tappings:—

May 12th, 1867, 4 gals.; June 4th, 4 gals.; June 16th, 4 gals.; Aug. 4th, 4½ gals.; Aug. 25th, 4 to 5 gals.; Sept. 4th, about 4 gals.

The tumor pressed upon the iliac vessels, and hence this rapid ascetic effusion. Not only this, the effect of these frequent tappings and the great loss of serum were manifesting themselves upon the patient's general health. She was, however, in the best condition for an operation, the morning of which the patient was ordered to remain in bed. The bowels and bladder having been voided, and the patient placed upon a common pine table, assisted by Prof. Powell, Drs. C. G. Smith and Rutter, the operation of Gastrotomy, for the removal of the tumor, was performed Sept. 4th, 1867. Although she was tapped Aug. 25th, the abdomen was considerably distended with fluid at the time of the operation.

Operation:—An incision through the skin and superficial fascia, from a point two inches above the symphysis pubis to a point one inch and a half above the umbilicus; hemorrhage having ceased, and the transversalis fascia and peritoneum punctured with a grooved director, a probe-pointed bistourie was introduced, and the small opening enlarged sufficiently to introduce the forefinger, which guarded the bistourie as it completed the incision to the above-mentioned points. There was considerable oozing at this stage, but it soon ceased. As the sides of the opening were pulled aside, a firm modena-colored tumor presented itself. The larger portion was on the left of the median line. It (the tumor) reached as high as the umbilicus. A pedicle three-fourths of an inch in length held it to the broad ligament. About the junction of the upper fourth with the lower three-fourths, the tumor was somewhat closely adherent to a coil of the small intestine. Upon further examination, the second tumor, from two inches to two inches and a half long, was found growing from the body of the womb. A third tumor, one-half inch in length, was located at the left angle of the fundus, near the entrance of Fallopian tube. Having adjusted the ecraseur, we endeavored to tighten the chain very gradually; but upon the most moderate break in the pedicle tissues, the blood welled up in profusion. The tumor was very easily separated from its pedicle—indeed too easily for the woman's safety and our own comfort. The pedicle was of a brittle nature, and its tissues readily broke under the pressure of the chain, whether the instrument was managed slowly or otherwise. When the tumor was separated from its pedicle, hemorrhage from the stump was so great, that the efforts of all of us were directed to that point. Silk ligatures were at first tried, but it was impossible to manage them on acccount of the firmness and shortness of the stump—the silk slipping from it, in spite of our best efforts to keep it in place. Finally, a needle, armed with a double ligature, was thrust through the stump, and tied on the side containing the bleeding vessels. There was considerable oozing from the adhesions as they were torn from the tumor, but from only one artery was there much hemorrhage. Just as we were about to ligate this vessel a new source of anxiety presented itself, in consequence of the interference of respiration by chloroform, probably assisted by the loss of blood. Of course, everything was to be dropped, and our energies and means directed to the remedying of this horrible condition. The face was livid; the parts about the corners of the mouth were already beginning to pale; inspiration had ceased. The patient was turned upon the side and back alternately; clots of blood and bloody serum rushed out upon the table—the intestines being held in place by the hand. In order to pull forward the tongue with a tenaculum, it required the strong iron handle of the pin-nippers to open the jaws, so firmly were they fixed. Water thrown upon the face, Spir. Ammon. held near the nose, artificial respiration, and brandy internally, resuscitated the patient. A ligature was applied to the remaining artery, the patient was again turned upon the side, to empty the abdomen of any remaining fluid, the ends of the ligatures were brought out at the lower end of the wound, and the latter closed with the hare-lip suture, which included the edges of the peritoneum. In a few places, where the abdominal walls were thick, silver wire was introduced, through the skin, between the pins, in order to close the wound as accurately as possible. Lint with simple cerate was placed over the line of incision, and over the lint a bandage. The patient, when removed from the table, was in very good condition. A little more brandy, containing Morph. Sulph. gr.ss. was given.

Sept. 4th, 4 P. M. Has vomited two or three times since the operation; has slept a few minutes at a time; pulse 96, soft and regular; expression bright and pleasant; patient in good spirits; skin moist; extremities warm; ordered Morph. Sulph. gr. $\frac{3}{4}$, in a little sherry; if thirsty, crushed ice.

Sept. 4th, 10 P. M. In good condition, but has vomited considerably since last visit; pulse 100; has slept a few minutes at a time. Ordered Morph. Sulph. gr. j., in sherry, and should it be vomited within half hour, use crushed ice till the stomach becomes settled; then give Morph. Sulph. gr. $\frac{3}{4}$; beef-tea, from one to four teaspoonsful, when she awakes through the night, provided she retained it.

Sept. 5th, 8 A. M. Pulse 102; skin soft, moist and warm; face a little swelled; no pain; slept some during the early part of the night; after 2 o'clock slept very well; vomited some through the early part of the night, when, at her urgent request, her husband gave her a half tea-cup of iced water, which seemed to settle her stomach, as she did not vomit afterwards, and whenever there was any tendency to vomit, a small drink of iced water would allay it. She vomited the grain dose of morphia, but not the second ($\frac{3}{4}$), and slept pretty well till morning.

Sept. 5th, 1 P. M. Pulse 96; drew off $1\frac{1}{2}$ pints high-colored urine.

Sept. 5th, 6 P. M. Bandages somewhat displaced; applied adhesive strips across abdomen to keep the lint in place; pulse 90; has slept two hours since last visit, and is feeling very comfortable.

Sept. 6th, 8 A. M. Pulse 114; has urinated two or three times since last night; slept from 8 last evening till midnight, without morphia; at 12 o'clock took a drink of iced water, and slept till 6 o'clock this morning; ordered chicken soup containing a small quantity of toast.

Sept. 6th, 5 P. M. Pulse 96. Being anxious to administer Tr. Ferri Chlor., but fearing it would irritate the bowels, only 15 gtt. were ordered, the dose to be increased should it agree with her.

Sept. 7th, 11 A. M. In pretty good condition, though not quite so well as she was yesterday. The dose of iron disagreed with her, and her attendants, very wisely, did not repeat the dose. It (iron) caused slight nausea, some vomiting, with pain through the intestines. She reports that the stomach swelled, (Tympanitis,) but is now subsiding; but very little tenderness; about midnight, she took Morph. Sulph. gr. $\frac{3}{4}$, but vomited it; the anodyne was repeated in half an hour, after which she was comfortable, and slept well; ordered her to continue chicken or beef soup; Morphia Sulph. when restless, or in any way uncomfortable.

Sept. 8th, 11 A. M. Awoke at midnight from colicky pains; took Morph. Sulph. gr. $\frac{3}{4}$, after which she slept till daylight.

Sept. 9th, 8 A. M. Passed very little urine last night; some burning in the hypogastric region; drew off one-half pint urine; dressed the wound; incision, for more than half its length, healed by first intention; a few drops of pus at the two points of exit of the ligatures, which are still held fast; considerable pain where the bladder is voided, in consequence of inflammation of its peritoneal lining; ordered warm water enema.

Sept. 9th, 5 P. M. Injection brought away no fecal matter; ordered Ol. Ricini ℥ss., at 6 P. M.

Sept. 10th, 8 A. M. Bowels moved at 1 A. M., bringing away a large quantity of dark, green, and very offensive matter; some pain during the operation of the bowels, but it subsided with the operation.

Sept. 12th. Removed the uppermost pin, the wound being perfectly healed at that part; a little suppuration about two or three of the pins.

Sept. 14th. Removed two pins from upper part of wound, supporting that part with adhesive plaster; ligatures still tight; the only suppuration worth mentioning is at the points where the ligatures pass through the wound.

Sept. 16th. Removed a pin from inferior end of wound.

Sept. 18th. Removed the two remaining pins.

Sept. 23rd. Patient up to-day for a short time; she wears a flannel bandage fitted to the abdomen, and retained in place by straps around the thighs.

Sept. 29th. Both ligatures came away to-day; at the upper orifice ʒss. of greenish pus came away. There being considerable tenderness, with some pain, on the right of the umbilicus, the patient was directed to remain in bed for a couple of days.

Oct. 1st. Paid last visit to-day. The wound has perfectly healed. The patient tells me that she feels as well as she ever did, except that she is yet a little debilitated. The bowels are regular, the urine passes easily and in good quantity, the appetite is good, and she sleeps well.

I have only time to give a rough description of the tumor. It was firm and heavy. The surface, over which ran rather large vessels, was, for the greater part, nodulated. The knife, as it passed through it, produced a creaking noise. It consisted of two elements, viz.: A fibrous material, so arranged as to form a greater or less number of cells or cavities, varying, in size, from a duck shot to a marble. These cavities, or cells, were filled with a colorless, jelly-like substance, of the consistence of very thin jelly. This cell contents was very easily pressed out; indeed, generally a touch seemed sufficient to cause it to disappear.

The sequel of this case occurs about two years from the date of the operation. Sunday, Aug. 22nd, 1869, about midnight, the husband of this woman came for me to visit her, at Evanston, in consultation with Dr. Geer, of that place. She was in labor, the pains having commenced about eight o'clock in the morning (Aug.

22nd.) The abdomen was much distended from the presence of two tumors, and a pregnant uterus at full term. The distension was so great, that, a month previously, a small opening appeared between the umbilicus and the pubis, in the line of the incision. The orifice discharged sometimes a dark, watery fluid, and sometimes a little pus. The integument around the fistule had ulcerated to the extent of the palm of the hand. Above the umbilicus there was another small opening. Two rather large tumors existed—one on each side of the uterus; but their bulk seemed so much incorporated with the womb's general enlargement, that their size could not be very definitely settled. The os tincæ could only be reached by introducing the whole hand, except the thumb, into the vagina. The mouth and neck of the womb were both soft and dilatable, but no part of the child could be touched. Nor were we able to detect the sounds of the fœtal heart. The amniotic fluid had come away during the afternoon. The pains came regularly, and the patient was generally in good condition. We left her till next morning, having first drawn off the urine.

Monday, Aug. 23rd. By introducing the hand, as before, we could touch the child's head. The womb occupied the same position as when we last saw her. Neither Dr. Geer nor myself were able, by pressing upon the abdomen, to cause the os tincæ to approach, in the least degree, the fingers of the right hand, in the vagina. The womb was firmly held in its abnormally high position. The fœtal heart sounds were again listened for, but in vain. The discharge from the vagina was of a greenish color, and somewhat offensive. We had feared hemorrhage. It was now our opinion that the child was dead; that the labor was progressing well under the circumstances, and that, as the patient was still strong, there was no indication for interference in any manner.

Dr. Geer, on Thursday, Aug. 26th, wrote me that the patient had been delivered of a still-born child, Tuesday, Aug. 24th; that there was no hemorrhage; that the cord was so soft that it would not bear any traction whatever, and that upon introducing the hand for the purpose of removing the placenta, he discovered quite a large bulge or platform, at the expense of the posterior wall of the womb. The placenta was fixed above this, in a *cul de sac.* The child was quite large; its cuticle was easily rubbed off; blebs were present in a few places. The tumors are now distinctly felt. There are three, viz.: one, 5 × 3½ in. on the left side of the uterus; another, 5 × 4½ in. on the right; a third, between the latter and the front of the womb. The third is small, and is, probably, an outgrowth of the second one. The patient tells me that, five months ago, the tumors were about the size of an egg. Several coils of intestines seem to be adherent to the walls of the abdomen, over which the skin and fascia are very thin. The womb did not contract very readily after the removal of the placenta. The child being dead and the fœtal circulation no longer going on, the vessels supplying the placenta and child became plugged up before delivery; hence, our expectations in regard to hemorrhage were not realized. Several months after delivery, the patient called to see me. The tumors were reduced in size about one-half.

*No.* 112 *Randolph Street.*

